# Husbands’ participation in birth preparedness and complication readiness plan in Kucha district, Gamo Zone, Southern Ethiopia

**DOI:** 10.1371/journal.pone.0261936

**Published:** 2021-12-28

**Authors:** Teklemariam Gultie, Zinash Tanto, Wubshet Estifanos, Negussie Boti, Barbora de Courten

**Affiliations:** 1 Department of Midwifery, College of medicine and Health Sciences, Arba Minch University, Arba Minch, Ethiopia; 2 Department of Nursing, Arba Minch College of Health Sciences, Arba Minch, Ethiopia; 3 School of Nursing, College of Medicine and Health Sciences, Arba Minch University, Arba Minch, Ethiopia; 4 School of public health, College of Medicine and Health Sciences, Arba Minch University, Arba Minch, Ethiopia; 5 Department of Medicine, School of Clinical Sciences, Monash University, Melbourne, Australia; Sabzevar University of Medical Sciences, ISLAMIC REPUBLIC OF IRAN

## Abstract

**Background:**

Birth-preparedness and complication-readiness (BPCR) is the process of planning for normal birth and anticipating the actions needed in case of an emergency. The involvement of husband during pregnancy helps a mother to make timely decisions to avoid delays. Identifying the level of husband involvement in Birth-preparedness and complication-readiness is very important, as husband is the major decision maker in household and health service related issue. However, there is no sufficient data in the Kucha district, which describes the level of husband involvement in Birth-preparedness and complication-readiness. Therefore, this study assessed the level of husband involvement in birth preparedness and complication readiness in Kucha District, Gamo Zone, Ethiopia.

**Methods:**

Community-based cross-sectional study was conducted on 421 husbands whose wife gave birth within the last 12 months at Kucha District using simple random sampling technique. Data was collected using a pretested interviewer-administered questionnaire by trained data collectors. Binary and multivariable logistic regression with odds ratios along with the 95% confidence interval analysis were employed to find factors associated with the level of husband involvement. A p-value <0.05 with 95% confidence level used to decide statistical significance.

**Results:**

Data were collected from 421 study participants. One hundred twenty-seven (30.2%) were involved in birth preparedness and complication readiness plan. Participants who had at least secondary school education AOR = 3.1, CI (1.84–5.23), had at least four antenatal care visits AOR = 4.91, CI (2.36–10.2), and live more than five km from the health care facility AOR = 2.35, CI = 1.40–3.96) were involved in birth preparedness and complication readiness plan.

**Conclusion:**

Husband**s’ i**nvolvement in birth preparedness and complication readiness was low. Husband’s higher educational level, high frequency of antenatal care, and long distance to the health facility were significantly associated with husbands’ involvement in Birth-preparedness and complication-readiness plan. Therefore, advocating for higher frequency of antenatal care and improving educational level are important to increase husbands’ involvement in birth preparedness and complication readiness plan.

## Introduction

About 295 000 women died during and following pregnancy and childbirth in 2017. The vast majority of these deaths (94%) occurred in low-resource settings. Sub-Saharan Africa and Southern Asia accounted for approximately 86% (254 000) of the estimated global maternal deaths in 2017. Sub-Saharan Africa alone accounted for roughly two-thirds (196 000) of maternal deaths [1]. In Ethiopia, the maternal mortality ratio is 412 per 100,000 live births [2].

More than two in three women (70%) in Ethiopia do not have access to health care [2]. Only 6% of the health facilities in Ethiopia provides basic emergency and obstetric care and 55% of the women do not use the available service due to delay in decision making to go to the health facilities [3]. Lack of access to essential health care exposes the women to experience obstetric complications such as unfavorable pregnancy outcome, maternal morbidity and mortality, premature birth, low birth weight and neonatal death [1,4].

A strategy to reduce maternal morbidity and mortality, birth preparedness and complication readiness plan was used as a prioritized intervention in order to reduce home delivery and decrease maternal morbidity and mortality [5]. Birth-preparedness and complication-readiness (BPCRP) is the process of planning for anticipating actions which will be needed in case of emergency, encourages women and households to make arrangements to give normal birth by reducing delays in reaching care once a problem arises [5,6]. It is crucial to decide on timely access to skilled maternal and neonatal services [7].

Finding from studies conducted in different African countries show that the majority of pregnant women were not having a birth plan [8,9]. For example, 35% of pregnant women were prepared for birth and related complications in Mbarara district of Uganda [10] and 27% in Ungogo northern Nigeria [9]. Despite different intervention to involve husband in participation in health care utilization by the government of Ethiopia and other stakeholders, most of the previous studies conducted in different parts of Ethiopia showed that less than 20% of women practice BPCR plan involving husband [11–13]. This is in particularly important as evidence from studies conducted in Ethiopia shows that husbands were the independent decision-maker on most family issues, like the decision on selecting a health facility or trained health professional to manage delivery [11,14,15]. These conditions make husbands a critical role player for the improvement of maternal and child health. Involvement of husband during pregnancy and delivery may help to support pregnant women be ready for any emergency obstetric care if any complications happens [16,17].

Study conducted among households targeting husbands with having at least one child of less than one year of age at Mekele town showed that variables like husbands’ awareness of postnatal danger signs, husband’s knowledge in birth preparedness, male involvement in antenatal care (ANC), educational status, economic status, and place of residence were factors associated with husband involvement in BPCR plan [8].

Therefore, the involvement of husband in BPCR during pregnancy, Labor, the postpartum period and its complication helps an expectant mother to make timely decisions to avoid delays that bring about complications that could result in morbidity or mortality and for achieving Sustainable Development Goals. However, little is known about the level of husband involvement in the study area which has different socio-economic and cultural differences with the previous studies. Therefore, this study aimed to assess the level of husband involvement in birth preparedness and complication readiness and associated factors.

## Materials and method

### Study area and period

This study was conducted in Kucha district, Southern Ethiopia, which is located 440 KM away from Addis Ababa and 172 KM away from the capital town of Gamo Zone, Arba Minch. In the district, there are 35 rural and one urban kebele (the lowest administrative unit in Ethiopian government system). In Kucha district there were eight health centers, 39 health posts, and 15 private clinics. This study was conducted from March 15/2018 to April 15/2018.

### Study design

A Community based cross-sectional study was conducted.

### Population

All husbands whose wife gave birth in the last 12 months in the selected kebeles of the district and live for more than six months in the district were included in the study. Those who were critically ill during the data collection period and husbands who were not living with their wives were excluded from the study.

### Sample size determination

The sample size for this study was determined using single population proportion formula (**n = (Zα/2)2 × p (1 − p)/d**^**2**^**)** [18] with the following assumptions; 95% confidence level, 80% power, 5% marginal error and 50.8% prevalence of husband involvement in birth preparedness and complication readiness which is taken from study conduct in Ambo town and 10% non-response rate [19]. The final sample size was 421.

### Sampling procedure

Simple random sampling technique was used to select the study subjects, the district has 36 kebeles and 7 kebeles were selected by lottery method, and then the calculated sample size was allocated proportionally to each kebele with consideration of the estimated number of husbands per each kebele. The sampling frame containing a list of all married women, who gave birth in the last 12months, was obtained from a family folder of health posts. In each health post there is a family folder that includes information about births, number of children, etc. we obtained the list of women who gave birth in the last one year before data collection period. Accordingly we allocate sample for each kebele (has one health post) based on the available data of women who gave both in the last one year. Unique identifier number was given for each households. Finally, from the list, women who gave birth in the last 12 months were selected randomly using computer generated technique and their husbands interviewed in a private place around their home. Those husbands who were not available at first visit were revisited for the second times and those who were not available were revisited for the third times.

### Data collection tool

The data collection tool was initially adapted from the survey tools used for maternal and neonatal health program which was developed by Johns Hopkins program for international education in gynecology and obstetrics (JHPIEGO) [14]. Additional information was included from published relevant literatures [9,14,15,20–23]. The questionnaire consists of information about socio-demographic characteristics, knowledge on danger signs during pregnancy, male involvement in birth preparedness and complication readiness plan. Initially, it was prepared in English. The English version of the questionnaire was translated to the Amharic language then to local language (*Gammotho*) and back-translated to English by language experts to check for its original meaning. The questionnaire was pre-tested on 5% of the total sample in Kucha town which was not included in the main data collection.

### Data collection procedures

The data collected by trained data collectors using face-to-face interview method of data collection. A total of eleven diploma holders’ clinical nurses and four health officers were recruited for the data collection and supervision of data collection process respectively. Both data collectors & supervisors were given a day-long intensive training on the data collection techniques, research purposes and research ethics.

### Data quality management

The questionnaire was pre-tested on 5% of sample size in Kucha town which was not selected for the final study. Based on the pretest, the logical sequence, as well as skip patterns of the questions was modified. Moreover, the time needed to complete an interview and the total number of days needed for data collection was estimated. Appropriate training for data collectors and supervisors was given which includes but not limited to the data collection process and the contents of the questionnaire. The overall activity of data collection was supervised and coordinated by the supervisors. After data collection, the collected data were checked for its completeness by the principal investigator. Before and after data entry, data were checked for any missing and inconsistencies.

### Data processing and analysis

Data were entered into EpiData version 4.4 software and then exported to IBM SPSS version 24.0 statistical product and service solution (SPSS) for analysis. Then, the data were cleaned to check for errors, and missed values. Descriptive statistics using frequencies, percentages, mean and standard deviations were used to describe findings. Binary logistics regression was done primarily to check which variables had an association with the dependent variable. Multiple logistic regression analysis was conducted to identify the independent predictors of husband involvement in planning birth preparedness and complication readiness. Those variables that had a significant association in binary analysis were selected for multivariable analysis. Both crude and adjusted odds ratios (AOR) with the respective 95% confidence intervals (CI) was reported and interpreted.

### Operational definitions

Men involved in birth preparedness and complication readiness practice: men who are involved in at least in two Birth preparedness plan (a plan for skilled birth attendants, place of delivery, and arrangement of money for transport or other costs) [14].

Good knowledge about pregnancy danger signs: husband who spontaneously mentioned at least two danger signs of pregnancy. Poor knowledge about pregnancy danger signs: husband who did not spontaneously mention two danger signs of pregnancy [24]. The key danger signs during pregnancy are severe vaginal bleeding, swollen hands/face and Blurred vision [7].

Birth-preparedness and complication-readiness (BPCRP): is the process of planning for anticipating actions which will be needed in case of emergency, encourages women and households to make arrangements to give normal birth by reducing delays in reaching care once a problem arises [5,6].

### Ethics approval and consent to participate

Before the study conducted, ethical clearance obtained from the ethical review committee of Arba Minch University, College of Medicine and Health Sciences institutional review board (IRB). Written informed consent obtained using standard disclosure procedures. Individual identifiers were removed to maintain the anonymity and confidentiality of the information. Interview was conducted in a private place where the audio and visual privacy were maintained.

## Result

### Socio-demographic characteristics of study participants

A total of 421 husbands were participated in this study with a 100% response rate. The mean age of the respondents was 34.11±5.5 years. Around half, 48.9% of them had age range of 30 to 39 years old. Regarding their ethnicity, the majority (72.7%) were Gamo and 102(24.2%) of them were government employees (Table 1).

**Table 1:** Socio-demographic characteristics of respondents in Kucha district, Gamo zone, southern Ethiopia, 2018.

Variables	Category	Frequency	Percent
Age in years	30–39	206	48.9
	40–49	215	51.1
Ethnicity	Gamo	306	72.7
	Gofa	14	3.3
	Wolaita	46	10.9
	Amhara	55	13.1
Husbands’ educational level	No formal education	27	6.4
	Primary education	161	38.2
	Secondary and above	233	55.4
Wife’s educational level	No formal education	56	13.3
	Primary education	221	52.5
	Secondary education	93	22.1
	College and above	51	12.1
Occupation	Government employee	102	24.2
	Private employee	13	3.1
	Merchant	77	18.3
	Daily laborer	79	18.8
	Farmer	150	35.6
Religion	Protestant	233	55.4
	Orthodox	137	32.5
	Muslim	51	12.1
Source of monthly income	Yes	166	39.4
	No	254	60.6
Average monthly income in ETB	500–1000	34	20.5
	>1000	132	79.5
Place of residence	Rural	324	77.0
	Urban	97	23.0
Number of wives currently present	One	402	95.5
	≥two	19	4.5

Note: One Ethiopian birr (ETB) is equivalent to 0.023 USD.

### Reproductive health and transportation

Three out of four study participants didn’t arrange transportation for their wives. Thirty five percent of the participants travel more than five kilometer to reach the health facilities. Only 10(2.4%) of study participant’s wife made decision by themselves to seek health care services (Table 2).

**Table 2:** Reproductive health and transportation related information of respondents in Kucha district, Gamo zone, Southern Ethiopia, 2018.

Variables	Response	Frequency	Percent
Distance to reach health facility	Greater than 5 km	147	35.0
	Less than 5 km	274	65.0
Frequency of ANC visit	Twice	129	30.9
	Three times	126	30.1
	Four and above	163	39.0
Arrangement of transportation	Yes	102	24.2
	No	319	75.8
Place of delivery	Health facility	400	95.0
	Home	21	5.0
Decision maker to seek health care	Husband only	404	96.0
	Wife only	10	2.4
	Husband and wife	5	1.2
	Relatives	2	0.4
Mode of transportation used to reach to health facilities	Ambulance	261	62.0
	Private car	14	3.3
	On foot	146	34.7

### Knowledge of obstetrics danger sign during pregnancy

Almost all of the study participants were aware of that severe headache and blurring of vision was one of the danger sign during pregnancy. Three out of four (72.4%) study participants were aware that leakage of amniotic fluid as a sign of obstetrics danger signs. Nine out of ten (92.1%) had a good level of knowledge about the danger signs during pregnancy (Table 3).

**Table 3:** Knowledge of obstetric danger signs during pregnancy among husbands in Kucha district, Gamo zone, Southern Ethiopia, 2018.

Variables	Response	Frequency	Percent
Vaginal bleeding	Yes	362	86.0
	No	59	14.0
Severe headache and Blurring of vision	Yes	420	99.8
	No	1	0.2
Leakage of amniotic fluid	Yes	305	72.4
	No	116	27.6
Convulsion	Yes	392	93.1
	No	29	6.9
High fever	Yes	387	91.9
	No	34	8.1
Persistent nausea and vomiting	Yes	353	83.5
	No	68	16.5
Swollen of hands/face	Yes	367	87.1
	No	54	12.9
Absent or decreased fetal movement	Yes	380	90.2
	No	41	9.8
Overall level of knowledge	Poor knowledge	33	7.9
	Good knowledge	388	92.1

### Level of husband involvement in BPCR

Less than one-third of husbands, 127(30.2%) were involved in planning birth preparedness and complication readiness (Table 4).

**Table 4:** Components of birth preparedness and complication readiness in Kucha district, Gamo zone, Southern Ethiopia, 2018.

Variables	Category	Frequency	Percent
Saving money	Yes	122	29.0
	No	299	71.0
Selection of skilled care provider	Yes	20	4.8
	No	401	95.2
Plan for a place to give birth	yes	174	41.3
	No	247	58.7
The arrangement of means of transportation	Yes	102	24.2
	No	319	75.8
The arrangement of blood donor in case of complication	Yes	7	1.7
	No	414	98.3
Overall husband involvement	Involved	127	30.2
	Not involved	294	69.8

### Determinants of husband involvement in birth preparedness and complication readiness

In the bivariate analysis, significant association was observed between husband involvement in birth preparedness and complication readiness and the educational levels of husband, the educational level of wife, knowledge on danger sign of pregnancy, frequency of ANC visits, if delivery was assisted, if arrangement for transportation to the health facility were made and distance to reach health facility.

After adjusting for the effect of confounding variables using multivariable logistic regression, educational level of husbands, number of ANC visits, arrangement for transportation and distance to health facility were associated with husband involvement in birth preparedness and complication readiness.

Husbands who had secondary and higher education were 3.1 times more likely AOR = 3.1, CI (1.84–5.23) to be involved in birth preparedness and complication readiness plan than those who had primary and below educational level. Husbands whose wife attended four and more ANC visits were 4.91 times more likely AOR = 4.91, CI (2.36–10.2) to be involved in birth preparedness complication readiness plan than those their counterparts. Families who lived far from a health care facility with a distance greater than 5km were 2.35 times more likely AOR = 2.35, CI = 1.403–3.96) involved in birth preparedness and complication readiness plan than those who live in less than 5 km distance from health facilities. Husbands who have arranged for means of transportation previously were 2.9 times more likely AOR = 2.9, CI (1.62–5.2) to be involved in planning birth preparedness and complication readiness than their counterparts (Table 5).

**Table 5:** Factors associated with Husband involvement in Birth preparedness and complication readiness in Kucha district, Gamo zone, Southern Ethiopia, 2018.

Variables	Category	Husband Involvement		COR (95% CI)	AOR (95% CI)
		Involved	Not involved		
		n (%)	n (%)		
Educational level	Primary and below	39(9.3)	148(35.2)	1	1
	Secondary and above	88(20.9)	146(34.7)	4.7(3.01–7.33)	3.107(1.84–5.23) *
Wife’s educational level	Primary and below	52(12.4)	225(53.4)	1	
	Secondary and above	75(17.8)	69(16.4)	2.28(1.47–3.55)	1.12(0.59–2.11)
Knowledge on danger sign of pregnancy	Yes	125(29.7)	263(624)	7.75(0.47–126)	3.79(0.16–88.50)
	No	2(0.5)	31(7.4)	1	1
Number of ANC follow up	Twice	12(2.9)	117(27.8)	1	1
	Three times	31(7.9)	95(22.6)	3.25(1.95–5.40)	2.66(1.52–4.65) *
	Four and Above	84(20.0)	79(18.8)	10.6(5.45–20.70)	4.91(2.36–10.20) *
Distance to reach health care facility	Greater than 5 km	68(16.2)	79(18.8)	3.13(2.03–4.84)	2.35(1.40–3.96) *
	Less than 5 km	59(14.0)	215(51.1)	1	1
Arrangement of transportation	Yes	80(19.0)	22(5.2)	5.49(3.29–9.15)	2.9(1.62–5.20) *
	No	47(11.2)	272(64.6)	1	1

Note

* = p-value<0.05.

## Discussion

The main finding of this study is that only one-third of husbands were involved in planning a birth preparedness and complication readiness. Husband educational status, number of antenatal care visits and living more than 5 KM from health care facility were significantly associated with husband involvement in birth preparedness and complication readiness.

The level of husband involvement in birth preparedness and complication readiness was 30.2% (95% CI: 25.8–34.6%). This is lower than studies conducted in rural Bangladesh, Endarta district, Ambo town and Wolaita Sodo town [15,19,20,25]. This difference might be due to socio-demographic and socio-economic differences such as low level of educational and economic status in the area of Ethiopia where study took place, which would contribute for low-level involvement in preparedness for birth. However, this finding is higher than the study done in southern Ethiopia and two studies conducted in a rural hospital of Rwanda [14,21]. This discrepant finding of our study and the one from Gamo Gofa zone could be related to the time of the study. Our study was done during the time the Ethiopian Governments gave special emphasis on health extension program to create awareness for rural communities on maternal health-related issues, in which the women and their family had sufficient opportunities to hear about the important components of birth preparedness and complication readiness. The free access for ambulance by the government may have also contributed to higher involvement in a birth plan as means of transportation is one components of birth preparedness and complication readiness plan. This finding implies that despite the improvement of husband participation in the birth preparedness plan it needs further exploration of the reason for low participation.

In this study, husbands’ educational level was one of the significant factors in promoting husband involvement in birth preparedness and complication readiness, Husbands who attend secondary and higher level of the school were involved in birth preparedness and complication readiness plan than those who attend primary school and below. This finding is supported by the study from northern Nigeria and Ambo Ethiopia [9,19] which indicate that the health-seeking behavior and involvement in health care decision making is improved with higher level of education of husbands. This finding implies that education is a key for averting many problems including behavioral changes and in addition to increasing access to universal education it is important to increase the level of education in the society.

The frequency of ANC visits was a significant predictor for husband involvement in birth preparedness and complication readiness plan. Husbands whose wife had four and more ANC visits were more involved in birth preparedness and complication readiness plan than their counterparts. This finding is similar to a study conducted in Wolaita Sodo town, Southern Ethiopia [26]. This finding indicates the need to increase the frequency of antenatal care. This can be achieved if the Ethiopian Ministry of health implement the new antenatal care guideline for positive pregnancy by world health organization.

Distance to the health care facility was one of the significant factors affecting husband involvement in BPCR plan. Husbands who live far from a health care facility with a distance greater than 5 km were more involved. This finding is consistent with the studies conducted in Wolaita Sodo town [26]. It is likely that husbands located less than 5km from health center were less concerned about birth complications as they live closer to the health facility. This study showed that there is a significant association between prior arrangements of means of transportation with husband involvement on BPCR plan. Accordingly, husbands who were arranging means of transportation before delivery were more likely to be involved in planning birth preparedness and complication readiness. This might be due to the assumptions that being ready by planning and arranging one component of BPCR will prevent the delay in reaching health care facility.

This study might be exposed for recall bias due to the reason that information was collected retrospectively. However, to minimize the recall bias we reduce the duration of last birth to one year. The other limitation as the study is cross sectional it is not possible to establish a cause effect relationship between the independent variables and outcome variable. Even though it is based on the reference of previous research, the measurement of involvement in birth preparedness and complication readiness and the level of knowledge it was difficult to compare with some studies.

## Conclusion

Husband’s involvement in BPCR is low in the study region of Ethiopia. Husband’s level of education, the number of ANC visits, distance to the health facility and pre-arrangement of transportation were significant factors associated with the husband’s involvement in BPCR.

Therefore, investing on antenatal care visits is crucial including the frequency of the care as it increases exposure with health-related messages including birth preparedness and complication readiness plan. The more information women get from health care provider the more likely to they are to convince their husband to be involved in the health care decisions. Improving education level of men in the community is important to improve the involvement of them in the BPCR plan.

## Abbreviations

ANC: Antenatal Care
AOR: Adjusted odds ratio
BPCR: Birth Preparedness and Complication Readiness (BPCR)
CI: confidence interval
IRB: institutional review board
MMR: maternal mortality ratio
JHEGO: Johns Hopkins program for international education in gynecology and obstetrics
COR: crude odds ratio
ETB: Ethiopian birr
SPSS: statistical package for social sciences
